# Harvest locations of goose barnacles can be successfully discriminated using trace elemental signatures

**DOI:** 10.1038/srep27787

**Published:** 2016-06-13

**Authors:** Rui Albuquerque, Henrique Queiroga, Stephen E. Swearer, Ricardo Calado, Sérgio M. Leandro

**Affiliations:** 1 Centro de Estudos do Ambiente e do Mar (CESAM) & Departamento de Biologia, Universidade de Aveiro, 3810-193 Aveiro, Portugal; 2 MARE – Marine and Environmental Sciences Centre, ESTM, Instituto Politécnico de Leiria, 2520-641 Peniche, Portugal; 3 School of BioSciences, University of Melbourne, Parkville, Victoria 3010, Australia

## Abstract

European Union regulations state that consumers must be rightfully informed about the provenance of fishery products to prevent fraudulent practices. However, mislabeling of the geographical origin is a common practice. It is therefore paramount to develop forensic methods that allow all players involved in the supply chain to accurately trace the origin of seafood. In this study, trace elemental signatures (TES) of the goose barnacle *Pollicipes pollicipes*, collected from ten sites along the Portuguese coast, were employed to discriminate individual’s origin. Barium (Ba), boron (B), cadmium (Cd), chromium (Cr), lithium (Li), magnesium (Mg), manganese (Mn), phosphorous (P), lead (Pb), strontium (Sr) and zinc (Zn) - were quantified using Inductively Coupled Plasma−Mass Spectrometry (ICP-MS). Significant differences were recorded among locations for all elements. A regularized discriminant analysis (RDA) revealed that 83% of all individuals were correctly assigned. This study shows TES can be a reliable tool to confirm the geographic origin of goose barnacles at fine spatial resolution. Although additional studies are required to ascertain the reliability of TES on cooked specimens and the temporal stability of the signature, the approach holds great promise for the management of goose barnacles fisheries, enforcement of conservation policies and assurance in accurate labeling.

European regulations (EC 1224/2009 and EC 1379/2013) on seafood traceability state that consumers are entitled to be truthfully informed about fishery and aquaculture products, and require that “*all lots of fisheries and aquaculture products shall be traceable at all stages*” and labeled to include “*the commercial designation, the scientific name, the relevant geographical area and the production method*”. Nonetheless, adequate labeling of seafood products is frequently absent, opening an opportunity for fraud due to the high market value that some seafood can reach in the trade^1^,^2^.

Trace elemental signatures (TES) imprinted in calcified structures have been increasingly used to investigate the environmental history of marine organisms^3^,^4^,^5^,^6^. Despite limited knowledge of the drivers of site-specific differences in calcified structures, TES have been used to track natal origins and dispersal of marine fish and invertebrate larvae^6^,^7^,^8^,^9^, detect migratory pathways^10^, classify habitats^11^, identify nursery areas^12^ and discriminate between wild and farmed fish^13^. Additionally, this methodology has been successfully employed to delineate geographically distinct stocks of fishes^14^,^15^, bivalves^16^,^17^ and crustaceans^18^.

The rationale of using TES to describe the environmental history of individual organisms is based on the geographic variation in water chemistry and on the uptake of trace elements from the environment. In cirripedes the trace elements appear to be absorbed along with Ca^2+^ through epithelial cells and secreted on the expanding plate^19^. As calcified structures accrete incorporating these trace elements, they document in a complex process the physicochemical properties of the environment, allowing them to function as geographic markers^20^. Fish otoliths, bivalve shells and other metabolically inert biogenic structures are especially useful as they permanently record the environmental history experienced by each organism^21^ and can be efficiently analysed using Inductively Coupled Plasma-Mass Spectrometry (ICP-MS)^8^. A wide range of trace elements are usually found in calcified structures of marine species, the most common ones being barium (Ba), boron (B), cadmium (Cd), calcium (Ca), copper (Cu), chromium (Cr), magnesium (Mg), manganese (Mn), phosphorus (P), lead (Pb), strontium (Sr) uranium (U) and zinc (Zn)^6^,^7^,^21^. Although some of these elements appear to accrete in proportion to their concentration in the water^22^,^23^,^24^, others do not have concentration-dependent uptake (eg. P and Mg)^4^. Some authors^24^,^25^ suggest that other physicochemical properties of the environment (eg. temperature, salinity, pH, dissolved oxygen) can play a key role in how some trace elements incorporate into calcified structures^26^. Regardless, an in depth understanding of the underlying causes of variation in TES is not necessary to apply this approach as a natural tag of source location^14^,^27^.

The high demand for seafood products places stocks, such as those of the goose barnacle *Pollicipes pollicipes*, under considerable fishing pressure. This crustacean is the most heavily exploited intertidal resource along the Iberian Peninsula, due to its high market value and growing consumer demand^28^. Data on consumption and total market value in Portugal is scarce. However, within the small Berlengas Biosphere Reserve (BBR), the annual harvest is estimated to be ~16 tons, resulting in a first sale market income of approximately 7500 €.harvester.year^−1^ 29. By extrapolating this figure for the Southwest Alentejo and Vicentine Coast Natural Park (SAVCNP), and taking in account the number of harvesting licenses currently awarded, these two protected areas alone are worth nearly 1 million €.year^−1^. In Galicia and Asturias (Spain), where goose barnacles are also considered a delicacy, 323 and 44 tons were landed, worth 7.6 and 1 million € in first sale market in 2014, respectively^30^,^31^.

Thus the goose barnacle fishery is an important source of income for local fishers in western Portugal^28^, and there are concerns that this may result in overexploitation^32^. Management plans for the harvest of *P. pollicipes* in Portugal are recent and impose temporal closures, size limits and daily catch limits to both recreational and commercial harvesters. There are also specific harvest regulations within two of the most important harvesting areas in Portugal: the Berlengas Biosphere Reserve (BBR) and the Southwest Alentejo and Vicentine Coast Natural Park (SAVCNP) (Fig. 1). In the BBR the fishery is restricted to licensed professional harvesters (20 kg.harvester.day^−1^). Due to the insular nature of this reserve and its small size, access to harvesting sites is more difficult and surveillance more efficient. In the SAVCNP, along with licensed professional harvesting (10–15 kg.harvester.day^−1^) licensed recreational harvesting is also allowed, albeit with a lower individual catch limit (1 kg. harvester.day^−1^). Due to the relatively easier access from land at this location and length of the coastline, surveillance is considerably less effective and there is growing concern that illegal and unreported harvesting can be significantly higher than that recorded at the BBR, which ultimately may hasten the depletion of *P. pollicipes* stock^28^,^33^.

Given this risk, the objectives of the present study were to: i) explore the possibility of using TES to discriminate among populations of *P. pollicipes* along the Portuguese coast; and ii) assess its feasibility as a forensic approach for tracing the origin of goose barnacles harvested in Portugal and elsewhere.

## Results

From the eleven trace elements (^137^Ba, ^11^B, ^111^Cd, ^52^Cr, ^7^Li, ^24^Mg, ^55^Mn, ^31^P, ^207^Pb, ^88^Sr and ^66^Zn) quantified in *P. pollicipes* shells from the ten sites sampled on the Portuguese coast, Mg and Sr presented the highest ratios to Ca, while Cd and Pb showed the lowest ratios (Fig. 2). The ratios of all 11 elements showed significant differences among locations, according to univariate one-way ANOVAs (Cr *F* = 50.102 *p* < 0.0001; Mn *F* = 14.146 *p* < 0.0001; Zn *F* = 16.503 *p* < 0.0010; Cd *F* = 11.223 *p* > 0.0010; Mg *F* = 7.000 *p* < 0.0001; Ba *F* = 7.944 *p* < 0.0001; Pb *F* = 7.359 *p* < 0.0001; P *F* = 6.58 *p* < 0.0084; Li *F* = 9.494 *p* < 0.0124; Sr *F* = 11.000 *p* < 0.0041 and B *F* = 12.825 *p* < 0.0144). Individuals collected in Figueira da Foz showed the highest ratios in Li, P, Cr, Mn and Sr; Baleal exhibited the highest ratio in B; the highest ratios in Mg and Pb were found in Sines; and the highest ratio in Zn was found in Cape Raso, which also showed high values of Cd and Ba (Fig. 2).

All the 11 trace elements were included in the RDA model (Li, B, Mg, P, Cr, Mn, Zn, Sr, Cd, Ba and Pb) as all elements presented statistical significant differences (*p* < 0.05), with the first 3 canonical discriminant functions explaining 82.58% of the total variance. The first canonical function explained 50.91% of the variance and was positively correlated with Mn, Ba and Cd and negatively correlated with Cr, Li and P. The second canonical function explained 19.07% of the variance, being positively correlated with Ba, Cd and Li, and negatively correlated with Mg, Sr and P (Fig. 3). The third canonical function explained 12.6% of the variance, with a positive correlation with Mn, Pb and Sr, and negative correlations with Zn, P and Cr. The jackknifed discriminant functions provided a success rate of 83% in the classification of the goose barnacles to their original place of origin.

Barnacles collected from the three sites from the BBR were correctly classified in 87% of the cases (Berlenga 100%, Estelas 80%, Farilhões 80%), and although reclassification success at Estelas and Farilhões was lower than that at Berlenga, 3 of the 4 misclassified specimens were attributed to adjacent locations in the Archipelago: 1 of the 2 misclassified individuals collected from Estelas was assigned to Farilhões while the 2 misclassified individuals collected from Farilhões were assigned to Estelas. The reclassification success of the 30 individuals collected from the BBR, irrespectively of the site of collection, was thus 97% ( =29/30*100). Moreover, only 2 individuals from locations outside the BBR (Praia da Ursa and Cape Sardão) were erroneously assigned to this region (Farilhões and Estelas respectively), representing merely 2.8% of the collected specimens in the remaining locations.

Average reclassification success of the barnacles collected from the two sites in the SAVCNP was 85% (Sines 100%, Cape Sardão 70%). The three misclassified individuals collected from Cape Sardão were assigned to locations outside the park: 2 were assigned to Berlenga and 1 to Estelas. Therefore, irrespectively of the site of collection, classification success of the barnacles collected from the SAVCNP was also 85% ( =17/20*100). Although the SAVCNP region showed a lower overall reclassification success when compared to BNR, it is of relevance to note that only 2 specimens from exterior locations (Baleal and Praia da Ursa) were assigned to the region, which is only 2.5% of the remaining individuals (Table 1).

## Discussion

All the elements analysed in the capitula of P. *pollicipes* displayed statistically significant differences (p < 0.05) amongst locations. Mg, Sr and P were the most abundant elements (Fig. 2), as commonly recorded for calcified structures^4^,^7^,^18^,^34^. This is most likely because these elements are common and relatively abundant in seawater^35^. Nonetheless the 4 most discriminating elements were Cr, Zn, Mn and Cd, suggesting that the most common elements follow similar patterns of incorporation among locations, or that the incorporation rate is independent of their concentration in seawater.

The three locations within the BBR region (Berlengas, Estelas and Farilhões), exhibited intermediate concentrations of all elements when compared to the remaining locations. This is most likely because the BBR is offshore and away from major urban areas, making it less susceptible to inputs from both riverine and wastewater influences, which are known to have elevated concentrations of many trace elements^36^. The SAVCNP locations (Sines and Cape Sardão) exhibited trace elemental signatures distinct from each other, as Sines predominantly displayed higher elemental concentrations than Cape Sardão. The lead concentration found to be higher in Sines is probably related to the proximity of the commercial harbor of Sines, the largest deep water port in Portugal, and having in its facilities a petrochemical terminal and a coal power station^37^,^38^. Baptista *et al*.^39^, found Pb accumulation in biomonitors from Sines, and Santos-Echeandía *et al*.^40^ reported higher concentrations of Pb in particulate trace element concentration in Sines coastal waters.

The higher concentration of Cr in Figueira da Foz (Fig. 2), is possibly related to the proximity of a calcium hydroxide (limewater) factory and their waste and production emissions^41^. This may also have influenced Mn concentrations, along with riverine runoff 4. Cape Raso presents the highest concentrations in Zn, Cd and Ba, which are generally associated with anthropogenic inputs^42^, namely the direct influence of Tagus estuary, the highly urbanized metropolitan region of Lisbon and a wastewater treatment plant marine duct located less than 5 km south of Cape Raso. High concentrations of these elements have been previously reported to occur in surface waters^43^ and sediments^44^ in nearby areas. Whereas Praia da Ursa is approximately 10 km north of Cape Raso, its elemental signature is remarkably distinct, with lower concentrations of Zn, Cd and Ba. This finding suggests that this location is not under the same influence of anthropogenic inputs as Cape Raso. Similarly, Peniche and Baleal are also geographically close, but have clearly discrete signatures. The proximity of the Peniche sampling location to the city’s fishing harbor, shipyard docks and a wastewater treatment plant, may explain the higher concentration of Cr when compared to Baleal, hence contributing to a distinct signature. Furthermore, along with the above discussed influences, it is also possible that these well-defined differences between adjacent locations are driven by headland-promoted oceanographic divergences. The capes Carvoeiro, Raso and Sines are associated with recurrent filaments produced by upwelling circulations^45^, which are known to induce relatively persistent environmental gradients in water chemistry over distances of a few kms^46^.

The RDA resulted in a jackknifed reclassification success of 70–100%, depending on site, but irrespective of the spatial distance among locations. The overall success to identify the source of barnacles collected from the 10 sites along the 300 km of coast was 83% (Table 1). The high discrimination indicates that the sites where the goose barnacles were collected have distinct signatures. BBR is located only 11 km away from the nearest continental locations (Baleal and Peniche), yet RDA based on TES had enough discriminatory power to correctly classify 97% of the goose barnacles collected from this region, whereas just 2.8% of the individuals were incorrectly assigned to BBR. The reclassification success of individuals collected form SAVCNP, 85%, was lower than that obtained in the BBR. The lower discriminant power obtained for the two locations from the SAVCNP is presumably associated with the unique TES of Sines, as previously indicated. However, the technique still performed relatively well in that only 2.5% of the individuals from outside the SAVCNP were incorrectly classified here.

Thus the present work was successful in demonstrating that the goose barnacle capitulum can be used to assign individuals to their harvesting site, on geographic scales from kms to 10 s kms along the Portuguese coastline. Moreover, it is shown that the present approach holds the potential to successfully discriminate between marine protected and harvest regulated areas of BBR and SAVCNP and the remaining collection sites, through the screening of geochemical fingerprints in calcified structures. These results suggests that even subtle differences in physicochemical properties of water masses can lead to different elemental concentrations, further stressing the potential use of TES to successfully trace the geographic origin of goose barnacles. Shifts in water chemistry, which may be promoted by fluctuating oceanic circulation, anthropogenic inputs and coastline arrangement over extended periods of time, are reflected in shifts in TES^5^,^6^.

Several authors, however, have reported temporal variability in TES signatures, from timescales of weeks to years, in fishes^47^,^48^, gastropods^21^ and bivalves^6^, whereas others suggest that TES can be temporally stable over the same timescales in fish otoliths^12^ and bivalves^7^. Such temporal instability in the signature could prevent correct assignment of individuals to their location of collection. To minimize this risk, authorities enforcing the correct geographical labeling and law-compliant fishing of goose barnacles would need to sample locations more prone to fraudulent or abusive practices during each fishing season. Thus, by matching the specimens being surveyed with those sampled from the most relevant fishing locations during the same season, authorities can be able to control for any potential temporal variability in TES.

Another potential limitation of the application of TES as an effective technique to trace the origin of goose barnacles is that crustaceans discard their hard parts at molting events which enable them to grow, thus resetting any imprinted signature. As a consequence most studies applying TES to crustaceans have used the whole body^6^,^34^, integrating the elements contained in the soft tissues to minimize the potential resetting of the signature. However, in goose barnacles, the shell plates are not shed during the molt; rather they are maintained and increase in size, being accreted at the periphery over time^49^, consequently preserving imprinted TES over their lifespan. With this in mind, the present work aimed to develop and validate a tool for tracing the origin of goose barnacles using shell plates. For this purpose barnacles with similar RC length, hence similar age, were selected. Additionally, the largest lateral shell was used, integrating the past and present TES of goose barnacles.

Although this technique was able to fully discriminate amongst collection locations, increased sampling effort over larger spatial scales could result in greater overlap in geochemical fingerprints among sites and the high degree of small-scale spatial heterogeneity will make it difficult to improve discrimination by grouping into regions. Further research is need to fully resolve the geographical scale over which TES can be used to accurately resolve the harvest locations of goose barnacles along the Iberian Peninsula.

A final consideration on the use of TES for traceability purposes is the stability of the chemical profile after harvesting. As calcified structures as the ones employed in the present study are biologically and chemically inert^50^,^51^, the geochemical signature is not expected to change after fishing, as it has been reported to happen when employing other tools, such as fatty acid profiles^52^,^53^ or genetic techniques^2^,^54^. However, as goose barnacles are consumed cooked (commonly they are boiled in water for 1–2 min), specimens can be traded raw or already processed. In this way, future studies should investigate if this cooking procedure somehow shifts the chemical structure of the capitulum and its TES.

The method detailed here shows that TES is a valid tool to investigate the geographical origin of goose barnacles, since it can discriminate among origins located at small spatial scales (<10 km). The forensic implications of this finding are great, suggesting TES can be an important tool for fisheries management and conservation of goose barnacles, enabling the detection of illegal poaching or unreported fishing, and as a convenient method for adequate labeling of this important seafood item.

## Material and Methods

### Species biology

*Pollicipes pollicipes*, popularly known as goose barnacles, is a sessile marine cirriped crustacean that is relatively abundant in rocky intertidal and shallow subtidal substrate of heavily exposed shores, with a geographical distribution ranging from France to Senegal^55^. The breeding period of *P. pollicipes* along the coasts of France, Spain and Portugal extends from the beginning of spring to late summer^56^. The species has internal fertilization and a planktonic larval phase that lasts a minimum of three weeks^57^. Adulthood is reached at ~28 mm in length along the Rostro-Carinal (RC) axis^58^ and individuals live up to 6 years. Legal commercial size (>23 mm RC) is attained one year after settlement^56^.

### Study area and sample collection

The circulation of the Western Iberian Coast is mainly controlled by the interaction among the Iberian Poleward Current (IPC), the upwelling/downwelling circulation induced by along-shore winds, and the Western Iberian Buoyant Plume (WIBP)^45^. During autumn and winter the circulation is predominantly northward, with a strong surface signature of the IPC and a well-defined WIBP to the north of the Estremadura promontory responding quickly to changes in wind direction and intensity. During spring and summer the circulation is mainly southward over most of the shelf due to the dominant northerly, upwelling-favorable winds. During this period short-term (3–9 days) reversals of the circulation occur, responding to the direction and intensity of wind events.

**Figure 1:**
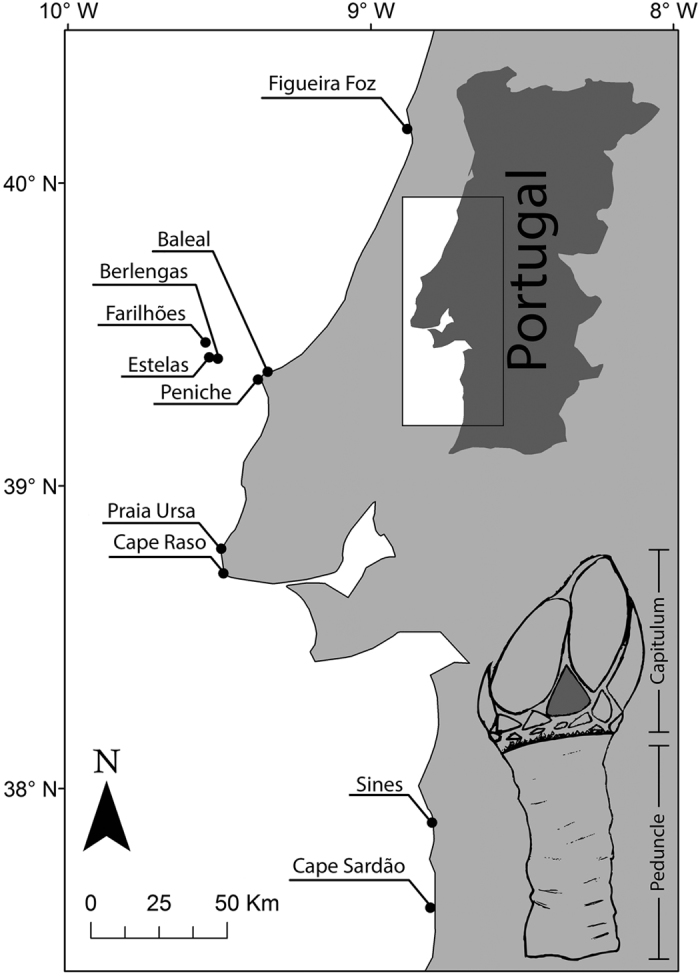
Sampling locations of *Pollicipes pollicipes* in the western coast of mainland Portugal: Figueira Foz (FF: 40.178598°N, −8.906513°W), Farilhões (FA: 39.473214°N, −9.545214°W), Estelas (ET: 39.424187°N, −9.533622°W), Berlengas (BG: 39.419675°N, −9.504889°W), Baleal (BA: 39.376588°N, −9.339759°W), Peniche (PE: 39.369378°N, −9.381259°W), Praia Ursa (PU: 38.792253°N, −9.49355°W), Cape Raso (CR: 38.710455°N, −9.486352°W), Sines (SN: 37.886998°N, −8.796691°W) and Cape Sardão (CS: 37.606126°N, −8.816595°W). Bottom right corner: drawing of a goose barnacle, the plate used in the analysis is shown in dark grey. The map was created using the software ArcGIS v9.2 www.esri.com/software/arcgis.

**Figure 2:**
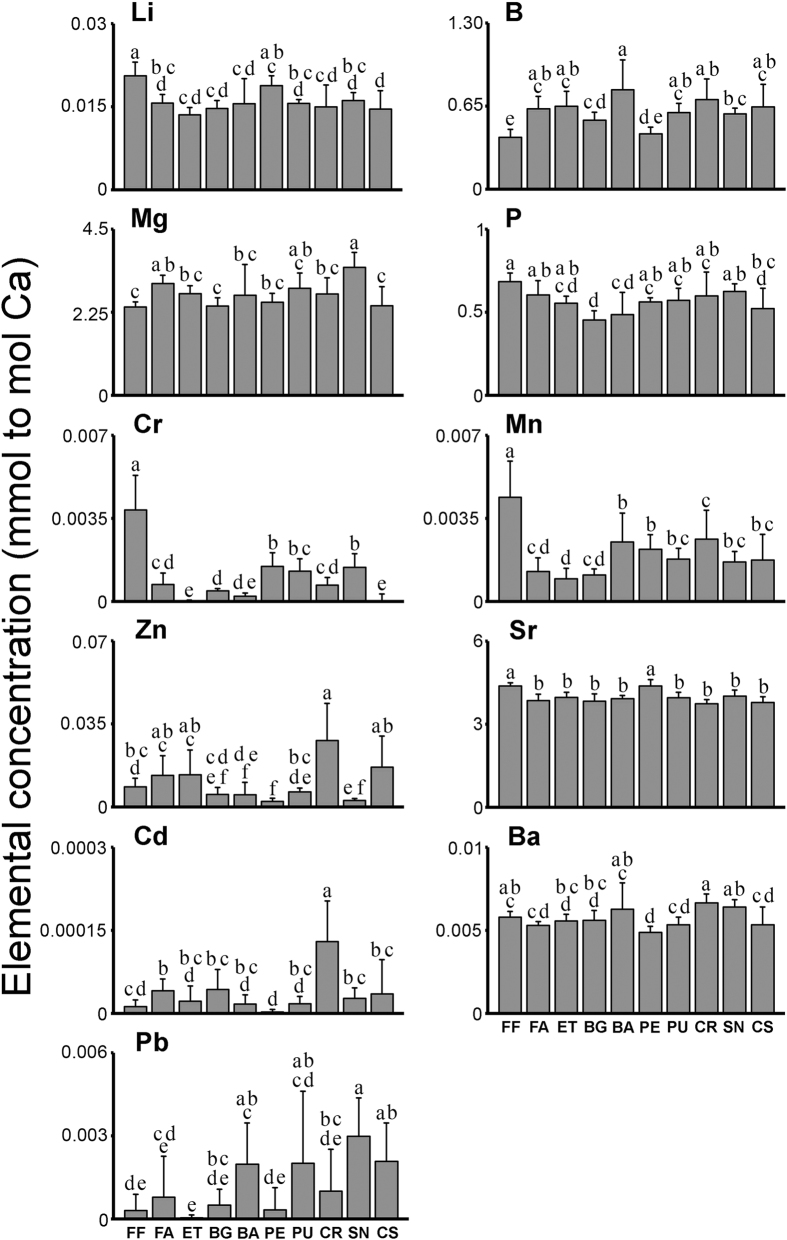
Ratios of trace elements to Calcium (X:Ca) concentrations (mmol to mol) (average ± SD) of *Pollicipes pollicipes* capitulum (the largest lateral shell) from ten locations along the western coast of Portugal. Significant differences (*p* < 0.05) among locations are noted with different letters.

**Figure 3:**
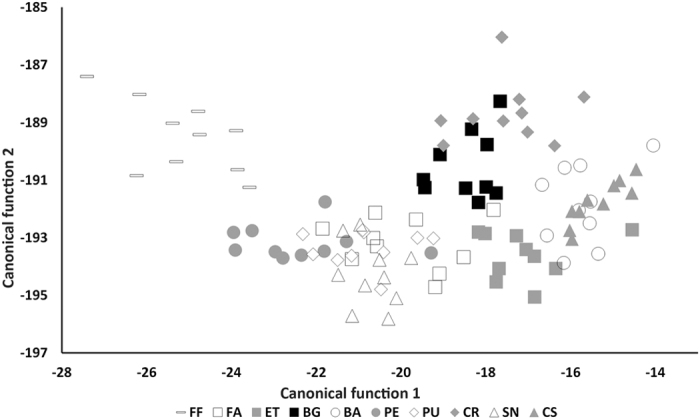
Canonical score plot of the regularized discriminant analysis (RDA) for the largest lateral shell of *P. pollicipes* by location: Figueira Foz (FF), Farilhões (FA), Estelas (ET), Berlengas (BG), Baleal (BA), Peniche (PE), Praia Ursa (PU), Cape Raso (CR), Sines (SN) and Cape Sardão (CS). Each point represents one individual. Sites within the same region are identified with similarly shaped symbols.

The present study was conducted along a 300 km section of the Portuguese coast, from Figueira da Foz to Cape Sardão (40°N to 37°N, Fig. 1). This region is characterized by a diversity of seascapes (capes, canyons, bays and estuaries), with long stretches of sandy shores alternating with rocky shores. *Pollicipes pollicipes* individuals were collected during June 2012 from ten different sites (Fig. 1). Sampling locations were selected based on the Portuguese regions with highest goose barnacles harvest^33^. Figueira da Foz, Berlengas Archipelago, the region around Praia da Ursa and Cape Raso, and the southwest coast around Sines and Cape Sardão. Additionally, two other sites, Baleal and Peniche, were also selected due to their proximity to the Berlengas Archipelago, in an attempt to differentiate continental and islands populations. All sites from the Berlengas Archipelago are located in the BBR, and the two sites in the southwest coast are located in the SAVCNP. At each site at least 5 different clusters of *P. pollicipes* were hand collected at distances ranging from 2 to 10 m from each other, stored in aseptic zipper bags, kept refrigerated during sampling and transported to the laboratory. Specimens were manually sorted in the laboratory in order to select individuals with a RC size >23 mm, which corresponds to the minimum legally allowed harvest size. These individuals were stored in labeled zipper bags and preserved at −80 °C within 2–3 h of collection for later processing.

### Sample preparation

In order to prevent contamination of collected samples a strict procedure was followed for all steps during analysis, using Milli-Q 18 MΩ water and reagents of certified trace metal purity: 30% H_2_O_2_, 99% NaOH of Suprapur^®^ grade and 60% HNO_3_ of Ultrapur® grade (Merck, Germany). Solutions were stored in previously acid leached HDPE bottles (48 hours in a 25:75% solution of HCl: Milli-Q). All plastic materials employed during processing were leached in acid solution (1N HCl fuming 37% Emsure^®^ grade) for 48 h, rinsed in Milli-Q water, allowed to dry under a laminar flow chamber and stored in zipper plastic bags. All other materials such as petri dishes and ceramic-tipped forceps coming into contact with the samples were acid leached in a 0.01 N HCl solution and thoroughly rinsed in Milli-Q water.

The preparation for ICP-MS analysis was performed in a class 100 clean room available at the Central Analytical Laboratory, University of Aveiro (certification ISO/IEC 17025). Ten individuals from each of the 10 sites were randomly chosen, the capitula were removed from the peduncle and the plates scraped of debris and transferred to plastic vials. In order to remove organic matter, the capitula samples were treated twice with 30 mL of 15% H_2_O_2_ buffered with 0.05 M NaOH in a microwave (400 W, 90 seconds), rinsed three times in 30 mL of Milli-Q water, and sonicated three times for 5 minutes. At the end of this process the water was removed and samples transferred to new vials and allowed to dry under a laminar-flow hood. The largest lateral plate of each capitulum was removed with ceramic-tipped forceps and digested in 15 mL acid-washed vials with 1 mL 60% HNO_3_. To avoid having Ca biasing the concentrations of the remaining elements, due to the much higher concentration already expected for this element, acid digested samples were diluted with Milli-Q water to a final concentration of 5% HNO_3_.

### ICP-MS analysis

Goose barnacle samples were analyzed for barium (Ba), boron (B), calcium (Ca), cadmium (Cd), chromium (Cr), lithium (Li), magnesium (Mg), manganese (Mn), phosphorous (P), lead (Pb), strontium (Sr) and zinc (Zn) at the Central Analytical Laboratory. Concentrations of ^137^Ba, ^11^B, ^111^Cd, ^52^Cr, ^7^Li, ^55^Mn, ^31^P, ^207^Pb and ^66^Zn were determined with a Thermo X Series Inductively Coupled Plasma−Mass Spectrometer (ICP-MS), equipped with a Burgener Mira Mist nebulizer, set up with a Peltier conical cooled chamber, quartz torch and silver shielded nickel cones, coupled to a Cetax ASX-510 auto sampler. Indium (In) and Terbium (Tb) were used as internal standards and the CeO/Ce ratio was kept below <2%. The concentrations of ^48^Ca, ^24^Mg and ^88^Sr were determined by a Jobin Yvon Activa M Inductively Coupled Plasma−Optical Omission Spectrometer (ICP-OES) equipped with a JY-AS500 auto sampler and a Burgener Mira Mist nebulizer.

### Statistical analysis

Prior to statistical analyses, concentrations of trace elements (μg l^−1^) were converted to element/Ca ratios (μmol/mol) in order to remove total mass effects^36^, and either natural log or fourth-root transformed in order to meet the normality and homogeneity of variance assumptions. Element/Ca ratios that met the assumptions were also transformed, in order to scale down concentrations to the range of the transformed ratios and place more emphasis on compositional differences among samples, rather than on the quantitative differences^59^. Differences in concentration of each element among sites were assessed using one-way analysis of variance (ANOVA), followed by Tukey’s HSD pairwise comparisons when significant differences were detected.

A regularized discriminant analysis (RDA) was performed to assess if TES can be used to infer the fishing location of surveyed specimens. Similarly to LDA, RDA is a constrained ordination tool that discriminates locations defined a priori and assesses the level of misclassification between sampled sites. However, RDA uses a regularization method to decrease the variance of the estimates of the discriminant function parameters when the number of variables measured (11 elements in our case) is similar to, or lower than, the number of observations in each group (10 observations per group in our case)^60^. A jackknifed procedure was applied to calculate classification success. This procedure ensured that the classification functions used to determine group assignment were unbiased, as each goose barnacle being classified is not used to construct the function, i.e., the estimates obtained indicate how well the RDA was able to correctly classify specimens from a new sample. Variables introduced in each model were selected following a forward stepwise analysis. To measure the effect of each element in the recorded differences between locations, Spearman correlation were calculated between all elements and the canonical axes. ANOVAs were performed with Statistica 12 (StatSoft, Inc., Tulsa, OK, USA), and multivariate analyses were conducted using JMP v10 (SAS Institute Inc. Cary, NC, USA).

## Additional Information

**How to cite this article**: Albuquerque, R. *et al*. Harvest locations of goose barnacles can be successfully discriminated using trace elemental signatures. *Sci. Rep.* **6**, 27787; doi: 10.1038/srep27787 (2016).

## Figures and Tables

**Table 1:** Classification success of jack-knifed RDA of *P. pollicipes* based on trace element signatures in the largest lateral shell from ten locations in the western coast of Portugal: Figueira Foz (FF), Farilhões (FA), Estelas (ET), Berlengas (BG), Baleal (BA), Peniche (PE), Praia Ursa (PU), Cape Raso (CR), Sines (SN) and Cape Sardão (CS).

		Predicted Locations										Total per Location	Reclassification success (%)
		FF	BA	FA	ET	BG	PE	PU	CR	SN	CS		
Original Locations	FF	9					1					10	90
	BA		7						2		1	10	70
	FA			8	2							10	80
	ET			1	8				1			10	80
	BG					10						10	100
	PE						9	1				10	90
	PU		1	1				7		1		10	70
	CR	2							8			10	80
	SN									10		10	100
	CS		2		1						7	10	70
												Average (%)	83
